# Dataset on mechanical properties of damaged fibre composite laminates with drilled vent-holes for resin-injection repair procedure

**DOI:** 10.1016/j.dib.2019.103912

**Published:** 2019-04-11

**Authors:** W.L. Lai, H. Saeedipour, K.L. Goh

**Affiliations:** a Newcastle Research & Innovation Institute (NewRIIS), 80 Jurong East Street 21, #05-04, 609607 Singapore; b Newcastle University, Faculty of Science, Agriculture and Engineering, Newcastle Upon Tyne, NE1 7RU, UK; c Republic Polytechnic, School of Engineering, Singapore

## Abstract

This dataset comprises the mechanical properties of pristine and barely visible impact damaged (BVID) carbon fibre reinforced epoxy composite (CFRP) laminates. The mechanical dataset describes the compressive strength, compressive strain at fracture, strain energy density for resilience and strain energy density to fracture of the CFRP laminates with different number of vent holes (namely 4, 5 and 6) in the respective pristine and BVID states. The vent holes were created in the laminate to facilitate the resin-injection repair process. The mechanical properties were determined from in-plane compression test of the CFRP laminates. Structure-related dataset, namely infrared thermographs and ultrasonic C-scan images of BVID and other damage features induced by mechanical drilling, are included for completeness.

**Table:** Specifications table

Subject area	*Engineering, Composite, Composite Repair, Mechanical Engineering*
More specific subject area	*Strength of materials, Vent hole application for composite repair*
Type of data	*Table, image, graph*
How data was acquired	*Infrared thermography (C-CheckIR, Automation Technology GmbH), Ultrasonic C-scan imaging system (Raptor, NDT Systems Inc), Mechanical tester (Instron 5982 and MTS Landmark Model: 370.25)*
Data format	*Raw, Filtered, Analyzed*
Experimental factors	*CFRP laminates (24 ply,* 100 mm by 160 mm*) were randomly selected into two groups (Mechanical state: Pristine or Damaged). Each group of samples were further sub-divided into 4 sub-groups (sample size/sub-group* = *3) (4 sub-groups are samples with (i) no holes, (ii) 4 vent holes, (iii) 5 vent holes and (iv) 6 vent holes). All samples were subjected to mechanical testing (in-plane compression test).*
Experimental features	*Quasi-static indentation (indentation depth* = 3 mm*) was carried out to induce damaged to the samples. Milling machine attached with a* 2-mm *diameter drill bit was used to create holes in the samples. Infrared thermography, ultrasonic C-scan imaging to derive the structural information. Mechanical testing (in-plane compression) was performed to derive the mechanical properties of the materials.*
Data source location	*Newcastle University in Singapore/Newcastle Research & Innovation* *Institute Singapore* *Republic Polytechnic, Singapore*
Data accessibility	*Data are available with this article*
Related research article	Reference 1 Lai WL, Cheah AYH, Ruiz RCO, Lo NGW, Kuah KQJ, Saeedipour H, et al. A simple portable low-pressure healant-injection device for repairing damaged composite laminates. Int J Mech Eng Educ 2017; 45. Reference 2 Rahman MAAS Bin, Lai WL, Saeedipour H, Goh KL. Cost-effective and efficient resin-injection device for repairing damaged composites. Reinf Plast 2018, doi.org/10.1016/j.repl.2018.11.001.

**Table:** 

**Value of the data** • The data is valuable because it contains important information concerning how drilling holes (for resin-injection repair) within the damage area may compromise the mechanical properties of the composite laminate. • The data could be valuable for informing materials engineer about (1) the material structure and its integrity after drilling holes (for resin-injection repair) in structural joints or assembly (2) the allocation of holes (i.e. number and spacing), within the BVID periphery for optimum strength recovery, (3) the performance and benchmarking of different types of drilling methods • The data could also direct future research, i.e. planning new and further experiments related to the issue of optimization of the resin-injection method for repairing multiple close-proximity damage sites as well as new analytical approaches related to the multiscale modelling of the response of damage after drilling holes in a material

## 1 Data

The data comprises the compressive properties of carbon fibre reinforced epoxy composite (CFRP) laminate with varying number of vent holes (no hole, 4, 5 and 6 vent holes) in pristine and barely visible impact damaged (BVID) states. Table 1 contains the data of the mechanical properties compressive strength, σ_U_, compressive strain at rupture, ε_U_, strain energy density for resilience, u_E_, and strain energy density to fracture, u_F_, calculated from the stress-strain curves derived from in-plane compression testing.

**Table 1:** Mechanical properties of CFRP laminates with different number of vent holes in pristine and damaged state.

	Pristine state				Damaged state			
	*σ*_*U*_	*ε*_*U*_	*u*_*E*_	*u*_*F*_	*σ*_*U*_	*ε*_*U*_	*u*_*E*_	*u*_*F*_
	MPa		MPa	MPa	MPa		MPa	MPa
No holes	304.2	0.0132	1.88	1.99	162.3	0.0111	0.66	0.69
	338.6	0.0154	2.21	2.81	159.0	0.0097	0.62	0.64
	275.9	0.0112	1.41	1.63	153.6	0.0100	0.62	0.63
4 vent holes	250.8	0.0108	1.31	1.41	136.7	0.0079	0.44	0.48
	258.4	0.0110	1.34	1.48	130.2	0.0064	0.39	0.42
	293.8	0.0115	1.64	1.71	147.5	0.0073	0.50	0.52
5 vent holes	250.0	0.0117	1.43	1.46	161.6	0.0076	0.56	0.59
	293.8	0.0115	1.64	1.72	163.9	0.0076	0.56	0.61
	222.0	0.0101	1.11	1.15	146.3	0.0068	0.47	0.49
6 vent holes	260.0	0.0119	1.48	1.58	126.3	0.0069	0.40	0.42
	262.3	0.0108	1.35	1.44	128.0	0.0067	0.39	0.42
	271.5	0.0101	1.31	1.40	132.6	0.0069	0.42	0.45

*σ*_*U*_ - compressive strength, *ε*_*U*_ - compressive strain at rupture, *u*_*E*_ - strain energy density for resilience, *u*_*F*_ - strain energy density to rupture.

**Fig. 1:**
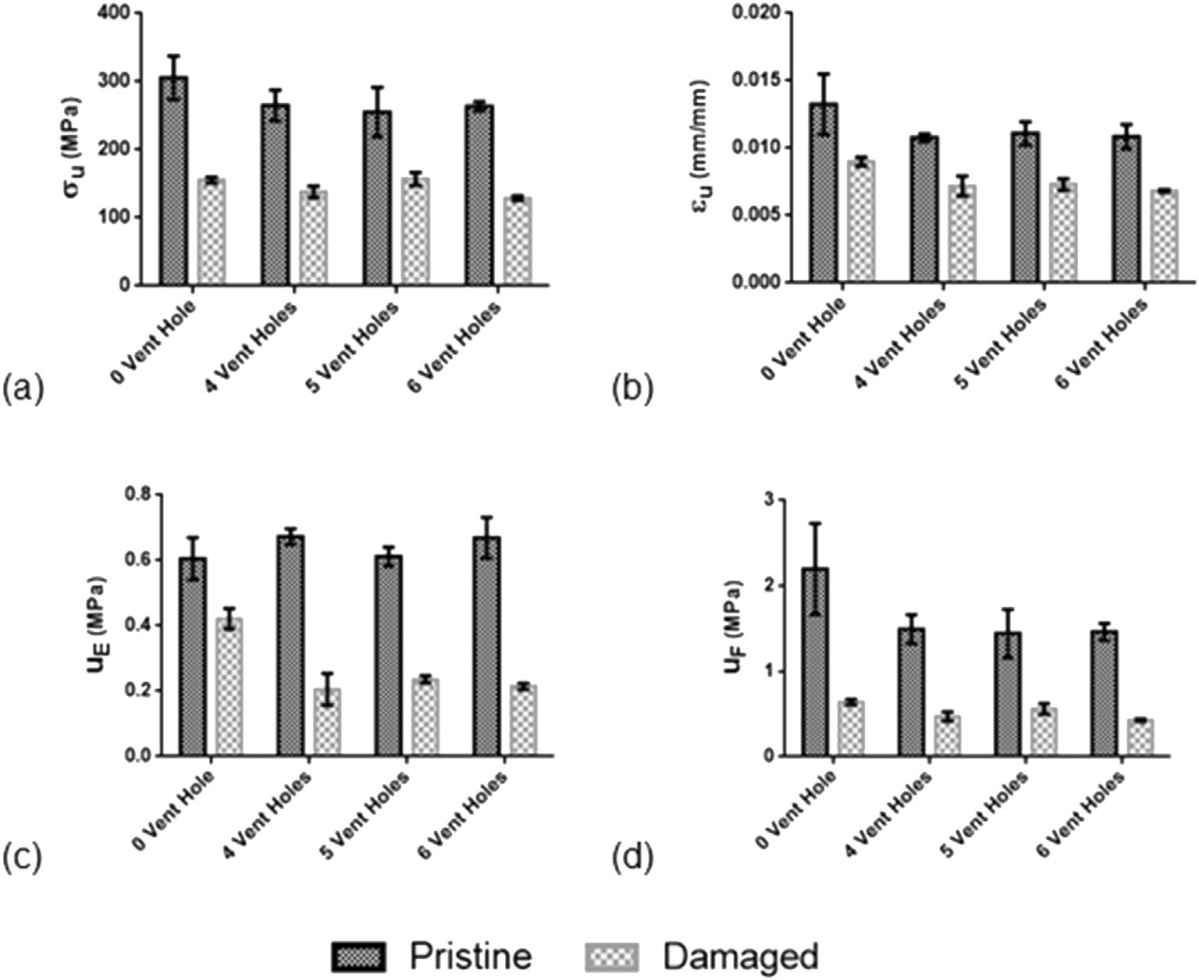
Bar charts of the mechanical properties of CFRP samples with varying number of vent holes in pristine and damaged state. (a) Compressive strength, σ_U_. (b) Compressive strain to rupture, ε_U_. (c) Strain energy density for resilience, u_E_. (d) Strain energy density to fracture, u_F_. Results are shown for sample mean and standard error of mean.

Fig. 1 shows the data of the mean and standard error of the mean value derived from the data (Table 1) of the mechanical properties for the four-level treatment with respect to number of vent holes.

**Fig. 2:**
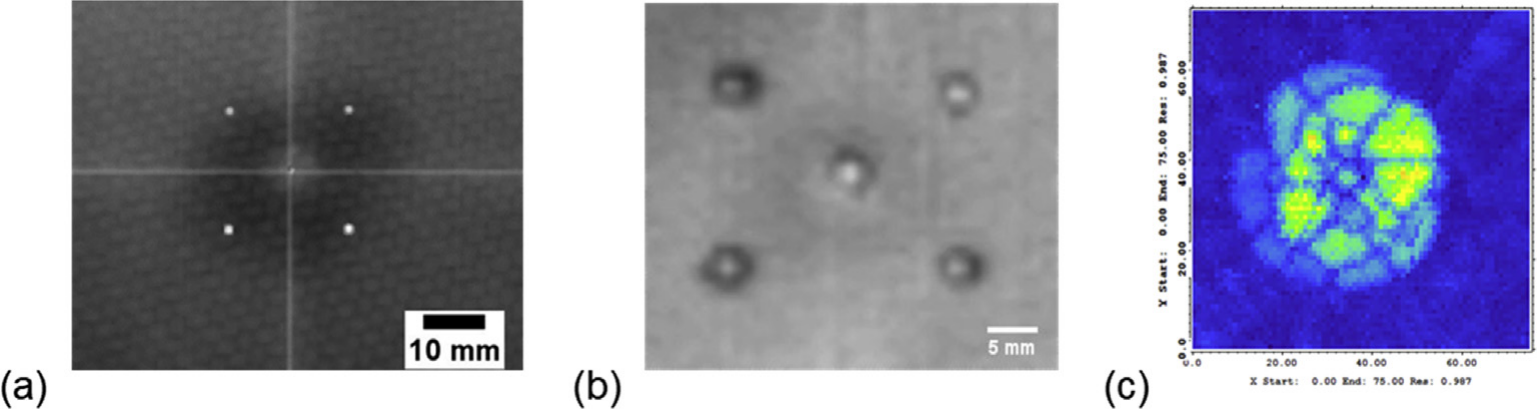
Images of the BVID CFRP laminates acquired by ultrasonic C-scan and Infrared thermography. (a) Thermogram of BVID in a CFRP laminate. (b) Magnified view of the thermogram in panel a, revealing the damage around the holes. (c) C-scan image of BVID in CFRP laminate.

Fig. 2 shows typical structural features of the CFRP laminates derived from infrared thermography and ultrasonic c-scanning, respectively. Panel a shows a BVID laminate sample in full view. The centre circular hole represented the resin-injection hole; the four white circular holes around the centre hole indicated the vent holes. These holes were drilled after the laminate was subjected to quasi-static indentation (QSI) to generate BVID [1], [2]. Panel b shows a close-up image of the damage around the respective vent holes. Panel c shows an ultrasound c-scan image of the BVID within the laminate specimen. The complete dataset of images resulting from this method can be found in a MS Excel file (Images_dib.xlsx).

## 2 Experimental design, materials and methods

### 2.1 Materials preparation

The data presented in section 1 were acquired from CFRP laminate specimens. A detailed account of the specifications of the CFRP laminate, the respective process of drilling vent holes and creating BVID using QSI is now described.

### 2.2 Mechanical test data

The mechanical test data was derived from 24-ply CFRP laminates, 100 mm (width) by 160 mm (length) by 4 mm (thickness), purchased from a local company (XPlas Pte Ltd, Singapore). Each laminate was made from unidirectional carbon fibre prepreg; the orientation of the 24 plies was described by a 4-ply repeat pattern given in standard notation as [45_o_/-45°/0°/90°]_6_. The outer layers of each 24-ply laminate were made from twill carbon fibre prepreg.

Of note, each laminate specimen was cut out from a larger piece of CFRP laminate (500 mm × 500 mm x 4 mm). In considering the possible variability in the inherent non-uniformities of the original CFRP laminate, a randomized procedure was used to identify and allocate samples to each treatment, i.e. pristine and damaged states. For mechanical testing, a total of 12 samples divided into 4 treatment groups (3 samples per treatment) were used to derive the mechanical properties related to the pristine condition, in the presence of varying vent holes, i.e. 0, 4, 5 and 6. Another 12 samples, divided into 4 treatment groups (3 samples per treatment) were subjected to BVID, to derive the mechanical properties in the damaged condition. The four different treatments corresponded to 0, 4, 5 and 6 vent holes. In all cases where vent holes were present, a resin-injection hole was also created.

The laminate specimens that were designated for damage treatment was subjected to QSI, according to a ASTM protocol for evaluating the damage resistance of a fibre reinforced polymer composite to a concentrated QSI force. In QSI testing, each laminate specimen was sandwiched between two steel blocks on the platen of a mechanical tester (Instron 5969, ITW Pte Ltd, Singapore). The indenter was hemispherical in shape (diameter 25 mm); it was mounted on a pair of grips on the test machine. The specimen was subjected to a pre-load of 200 N, at a displacement rate of 1 mm/min. Then, the indenter was driven into the specimen to a depth of 3 mm, at a displacement rate of 1.25 mm/min. This approach resulted in imparting an impact energy of about 12 J, which was within the range of energies associated with BVID, to damage the specimen.

Subsequently the dataset containing the mechanical properties of the CFRP samples was derived by subjecting the samples to in-plane compression test, following the prescribed protocol in ASTM D7137 [3]. Each sample was held firmly in the in-plane compression test fixture with all bolts tightened to 7 Nm using a torque wrench. Mechanical tester (MTS tester, Landmark Model 370.25) fitted with a 300 kN loadcell, was initially used for acquiring data from pristine samples. Of note, data from pristine samples showed that they failed at loads of 250 kN. Owing to logistic difficulties arising from the use of the MTS tester, and also because BVID samples fractured at much lower loads, the Instron 5982 tester (with a much lower loadcell capacity, namely 100 kN) was implemented for BVID samples. All samples were loaded in compression at a displacement rate of 1.25 mm/min until the samples ruptured.

Data of the load (F) and corresponding displacement (ΔL) was collected from the in-plane compression tests. The data was used to determine the corresponding stress (σ) and strain (ε). The σ was set equal to F/A, where A was the cross-sectional (normal to the loading axis) of the short edge of the laminate specimen. By measuring the respective width (w) and thickness (d) along the laminate at three different points the A was found by multiplying the average values of the respective w and d. The ε was set equal to ΔL/L_o_, where L_o_ was the original length (=160 mm), the long edge parallel to the loading axis of the laminate sample.

The analyzed data from the σ versus ε plots consists of compressive strength (σ_U_), compressive strain at rupture (ε_U_), strain energy density for resilience (u_E_) and strain energy density to rupture (u_F_). The σ_U_ and ε_U_ were identified at the point when the samples ruptured (F). The u_E_ was parameterized by the area under the curve from the origin (O) of the stress-strain graph to the end point of the linear region. The u_F_ was parameterized by the area under the curve from O to F.

### 2.3 NDT data

The data collected from non-destructive testing (NDT) methods, namely infrared thermography and ultrasonic C-scan imaging, was intended to detect for damage within the CFRP laminates [4], [5].

For infrared thermography, the damaged specimen was placed on a flat surface under a halogen lamp of the imaging system (C-CheckIR, Automation Technology GmbH, Germany). The halogen lamp heated the specimen to 50 °C for about 20 seconds. The thermograph, an image depicting the temperature variation (attributing to the material inhomogeneity within the laminates) on the specimen on the surface of the laminate, was recorded by an infrared camera.

With regard to ultrasonic C-scan, an ultrasonic C-scan system (Raptor, NDT Systems Inc, United States), comprising an ultrasound flaw detector, a manual X—Y string scanner and a 15 MHz contact probe was used for imaging the BVID. The intent was to compare the extent of the damage with that observed on the thermograph. The C-scan image was not useful for assessing damage around the holes in the CFRP laminates because the flaws smaller than 1 mm^2^ could not be observed.

As pointed out in the previous paragraph the dataset of the thermograph was used for assessing the extent of the BVID site. This information was then used to determine the positions of the vent holes in damaged specimens before the holes were created. To create the vent holes, first a circle of 50-mm diameter (covering the area of the BVID) was drawn, centered on the BVID. (The size of this circle was determined empirically, based on observation of all the BVID samples.) Then, the resin-injection hole was marked to the centre point of the circle. This point denoted the origin of the polar coordinate system for determining the positions of the vent holes. The vent holes were located on the circle at equal spacing with respect to the azimuthal angle.
