# Supplementary material for: Dataset on mechanical properties of damaged fibre composite laminates with drilled vent-holes for resin-injection repair procedure
Source: Data Brief. 2019 Apr 11;24:103912. doi: 10.1016/j.dib.2019.103912 (PMC6488824; doi:10.1016/j.dib.2019.103912)
Supplement: Supplementary file 1 — Multimedia Component 1 [file mmc1.doc]

Conflict of Interest and Authorship Conformation Form

Declarations of interest: none

- All authors have participated in (a) conception and design, or analysis and interpretation of the data; (b) drafting the article or revising it critically for important intellectual content; and (c) approval of the final version.
- This manuscript has not been submitted to, nor is under review at, another journal or other publishing venue.
- The authors have no affiliation with any organization with a direct or indirect financial interest in the subject matter discussed in the manuscript
